# CVD 103-HgR live, attenuated cholera vaccine strain viability in drinking waters from the US and Europe

**DOI:** 10.1038/s41598-021-92182-3

**Published:** 2021-06-23

**Authors:** R. Paul Duffin, Michael Delbuono, Kylie Nishioka, Paul Shabram, Amish A. Patel

**Affiliations:** Emergent BioSolutions, Inc., Gaithersburg, MD 20879 USA

**Keywords:** Live attenuated vaccines, Pharmaceutics

## Abstract

CVD 103-HgR live, attenuated oral cholera vaccine strain is indicated for single dose immunization against *Vibrio cholerae*, the causative agent for cholera. The vaccine packets containing buffer powder and lyophilized CVD 103-HgR are reconstituted in water and consumed. Studies were performed to explore the viability of CVD 103-HgR in drinking waters from common sources. CVD 103-HgR vaccine was reconstituted in bottled and tap waters from the United States and Europe, and viability was measured via colony forming units assay. Chemical analysis of select water samples was used to identify chemicals that have a negative effect on CVD 103-HgR viability. CVD 103-HgR titers were stable in all bottled waters tested, including purified bottled water, bottled spring water, and sparkling waters. However, tap water from certain cities in the US and Europe affected viability and are not compatible with vaccine. Water chemistry revealed that these tap waters contained copper, likely leached from copper plumbing. These studies give high confidence in the stability of CVD 103-HgR reconstituted in a variety of bottled waters. Waters containing copper, including tap water, should not be used to reconstitute CVD 103-HgR strain oral vaccine due to the common use of copper plumbing.

## Introduction

Cholera is an enteric disease caused by the Gram-negative *Vibrio cholerae* bacterium that is characterized by severe watery diarrhea, and vomiting that can lead to dehydration, shock, and death if left untreated^1^. There are several oral cholera vaccines available worldwide, the majority of which are whole-cell killed bacteria that require more than one dose^2^. PXVX0200 is a single dose live attenuated oral cholera vaccine containing the CVD 103-HgR vaccine strain of *V. cholerae* O1 Inaba 569B that is licensed under the tradename Vaxchora by the US Food and Drug Administration (FDA) and European Medicines Agency (EMA). Per the published instructions for use for Vaxchora vaccine^3,4^, the PXVX0200 vaccine is prepared by reconstituting the buffer and CVD 103-HgR active components in 100 mL of water. One dose of the vaccine consists of 4.5 g of buffer component (sodium bicarbonate, sodium carbonate, ascorbic acid, and lactose) and 2 g of the active component (lyophilized CVD 103-HgR, hydrolyzed casein, sucrose, ascorbic acid, and lactose) with a commercial specification of 4 × 10^8^ to 2 × 10^9^ colony forming units (CFU) per dose.

Live vaccine strains, such as CVD 103-HgR have the advantage of producing a long-lasting immune response with a single dose by mimicking natural infection^5^, but they can also show greater susceptibility to factors such as temperature and components in the vaccine mixture^6^, including the water used for reconstitution. Here, we describe a study exploring the compatibility of the cholera CVD 103-HgR strain with drinking water from different sources in the United States (US) and Europe in order to better characterize the vaccine and to ensure that the full potency is administered to the patient. Compatibility in this context is defined as maintaining viability of CVD 103-HgR, as measured by CFU/mL, within approved vaccine specification.

PXVX0200 has the primary role of preventing travelers from developed nations from contracting and spreading cholera to vulnerable populations. In developed nations, drinking water is regulated by government agencies based primarily on guidelines published by the World Health Organization that describe permissible limits of chemical and biological contaminants^7^. Drinking water in the United States (US) and European Union (EU) can be broadly divided into two categories: municipal (tap) water and bottled water. Municipal water is regulated by the Environmental Protection Agency (EPA) in the US^8,9^ and by the European Commission in the EU^10^. Bottled water is a specified type of drinking water that must meet additional standards and is regulated by the FDA in the US^11^ and by the European Commission in the EU^10,12^.

This report provides data from studies performed on the compatibility of CVD 103-HgR live, attenuation cholera vaccine strain with drinking water from various sources, including tap water and bottled water from the US and Europe.

### Tap water

The majority of public tap water is derived from surface and ground water, such as lakes, rivers, reservoirs, and underground aquifers. The water is processed through municipal water treatment plants by different chemical and physical methods depending on the source and quality of the water. Treatment plants use a combination of methods to physically remove particulate mass from the water which include flocculation and sedimentation with iron salts and alum, and filtration. Disinfection of the water most commonly relies on chemical treatment using chlorine, chlorine dioxide, or monochloramine which can remain in residual concentrations at the point-of-use and may result in biproducts^13^. Ascorbic acid, which is a major ingredient in the PXVX0200 buffer component, has been shown to effectively neutralize both chlorine and chloramine^14^. Pipes that carry the drinking water from the treatment plant to the point-of-use can also leach chemicals that are present when the water is collected from the tap^15^. The content of chemical compounds in tap water varies between municipalities that can be derived from the pre-treated water source, disinfecting and treatment methods, and drinking water infrastructure including household plumbing.

Several small tap water studies have been performed on compatibility of tap water with CVD 103-HgR active component during the development of the PXVX0200, including waters from three US cities (Miami, Redwood City, and San Diego) and four European cities (Bern, Milan, London, and Madrid). CVD 103-HgR viability and water chemistry experiments were performed on tap water samples from San Diego, Redwood City, and Madrid (see Table 2 in “Results” section). From these studies, candidate chemical compounds were identified and spiked into molecular biology grade water which was used to reconstitute PXVX0200 in order to investigate their effect on the viability of the CVD 103-HgR active component.

### Bottled water

Bottled water may be specifically labelled depending on qualities of the water such as the source, treatment method, and mineral content. In the US, bottled water may be labelled as artesian, mineral, sparkling, spring, or purified bottled water. Purified bottled water may be further defined by the method used to treat or purify the water, including distilled, demineralized, deionized, or reverse osmosis water^11^. In Europe, bottled water may carry the label of natural mineral water, spring water, and bottled drinking water^10^.

## Materials and methods

For all bottled water, tap water, and chemical spike studies, the PXVX0200 buffer component and the CVD 103-HgR active component were reconstituted in 100 mL of ambient temperature water (15–25 °C) per the package insert instructions. Viability (calculated as CFU/dose) was assessed immediately after reconstitution, and then at 15 and 30 min post-reconstitution. Water samples were tested in duplicate. Duplicates were performed by reconstituting the vaccine in 100 mL from two separate bottles of bottled water, or two separate 100 mL aliquots of tap water. Five samples from each reconstituted vaccine were plated for CFU analysis per time-point.

In all cases, reconstituted vaccine sample was diluted into chilled buffered peptone water (kept on ice) to achieve concentrations within the linear range of the CFU assay (200–40,000 CFU/mL). Samples were plated onto tryptic soy agar (TSA) plates (BioMerieux, M1040-IR) using the WASP Touch Spiral Plater (Don Whitely Scientific) or Autoplate Spiral Plating System (Advanced Instruments, Inc). Sample were diluted and plated using the 50 μL logarithmic mode, per plating system instructions. The theoretical lower limit of detection of the assay is 2 × 10^5^ CFU/dose. Colonies were quantified using the ProtoCOL 3 Plus automatic colony counting and zone measuring system (Synbiosis). Five TSA plates were inoculated using the spiral plater with 50 μL of sterile WFI quality water as a negative control for each experiment, with no more than 5 colonies/plate allowed.

Seven lots of CVD 103-HgR containing active component were used in these studies. Lot FA03 and lot 001 were produced in San Diego, and lots 054, 230, 231, 232, and 076 were produced in Bern, Switzerland. Buffer component lots were used interchangeably depending on availability.

All stability time-points were determined using the CVD 103-HgR active product reconstituted in chilled buffered peptone water (Hardy Diagnostics) per the SOP for the PXVX0200 vaccine stability program. Stability is defined as the ability of Vaxchora to maintain a CFU/dose titer above the lower limit of potency (4 × 10^8^ CFU/dose) after reconstitution in water.

### Tap water from the US and EU

An early study (July 2015) was performed to investigate the viability of CVD 103-HgR in tap waters from Miami (Florida), San Diego (California) and Redwood City (California). A second PXVX0200 tap water viability study was performed in May–June 2018 using tap waters that were obtained from Milan (Italy), London (UK), Bern (Switzerland), and Madrid (Spain) in Europe. Finally, fresh samples of tap water were collected in February–March 2019 from San Diego, Madrid, and Redwood City for re-testing using both CFU and water chemistry in order to correlate the effect of specific chemical compounds with the CVD 103-HgR active component viability. San Diego and Madrid tap water were selected because of observed effects on the viability of the CVD 103-HgR active component when used for reconstitution in the earlier studies, and Redwood City tap water was chosen as a control since CVD 103-HgR active component viability was not affected in the previous experiments. All samples were collected in 1L PETG bottles and shipped to San Diego where they were stored at 2–8 °C until testing.

### Tap water chemistry analysis

Chemical analysis panels were performed on aliquots of the San Diego, Madrid, and Redwood City tap water collected in February–March 2019 by EnviroMatrix Analytical, Inc in San Diego, CA. Tests included Total Metals by EPA 200 methods, Organochlorine Pesticides by EPA method 8081B, Organophosphorus Pesticides by EPA method 8141A, Volatile Organic Compounds by EPA method 8260B, and conventional chemistry parameters such as bicarbonate alkalinity and carbonate alkalinity by National Environmental Methods Index (NEMI) method 2320 B, ammonia as N (EPA method 350.1), chloride (NEMI method 4500-Cl B), residual chlorine and chloramines (NEMI method 4500-Cl G), specific conductance (NEMI method 2510B), methylene blue active substances (NEMI method 5540 C), total phenols (EPA method 420.1), and phosphorus (NEMI method 4500-P B, and 4500-P E). Additional testing was performed specifically for total organic carbon (NEMI method 5310B), fluoride (EPA method 300.0). Water chemistry methods were performed in singlicate.

### Tap water chemical compound spiking studies

Using the results of the water chemistry analysis, candidate chemical compounds were identified that could potentially affect the CVD 103-HgR active component viability. The chemicals were individually spiked into molecular biology grade water (MBGW) samples at levels that were 10-times the maximum contaminant level (MCL) allowed by the EPA regulations^9^, which was then used to reconstitute PXVX0200 buffer and active components. For example, the MCL for copper is 1.3 mg/L so for these studies copper was spiked into the MBGW to a create a solution with the final concentration of 13 mg/L copper. If the viability of CVD 103-HgR was affected at the 10X concentration, fresh samples were prepared at lower concentrations to determine the sensitivity of the CVD 103-HgR bacteria to the contaminant. 100 mL of the solution was used to reconstitute the vaccine, and samples were taken at 0, 15, and 30 min post reconstitution samples to test for CVD 103-HgR viability, as described above. The chemicals that were investigated were Bromodichloromethane (VWR, 100519-450), bromoform (SPEX CertiPrep, SXS-5555), dibromochloromethane (SPEX CertiPrep, SXS-1230), copper sulfate (RICCA Chemical Company, 2347-32), and fluoride (RICCA Chemical Company, R3171000).

A separate study was performed to investigate the viability of CVD 103-HgR active component when reconstituted in water spiked with sodium hypochlorite (Sigma-Aldrich, 239305) at 5, 10, and 100 mg/L.

### Bottled water from the US and EU

Purified bottled and bottled spring water brands in the US were selected based on the sales of the leading bottled still water brands according to the 2015 Statista Bottled Water in the U.S. dossier^16^. European brands of still and sparkling were chosen based on data 83% of bottled waters produced in Europe are labelled Natural Mineral Water according to the European Federation of Bottled Waters, a trade association representing the interests of the European bottled water industry. Detailed market share data on the most popular brands of bottled water in the EU were not available, so brands were chosen based on availability from two international retail chains, REWE (Germany) and Carrefour (France), including the store brands, because of the availability between countries. Representative bottled waters from different regions of Europe were chosen to ensure more thorough analysis.

Purified bottled water brands used for these studies were DASANI, Aquafina, Nestlé Pure Life, Glaceau Smartwater, Niagara, and Kirkland Signature. Bottled spring water brands used for these studies were Poland Spring, Deer Park, Ozarka, evian, Crystal Geyser, and Arrowhead.

European brands of still Natural Mineral Water used for these studies were Volvic and Evian (France), Acqua Panna (Italy), Henniez (Switzerland), REWE (Germany) and Carrefour (France). European sparkling water brands used for these studies were S.Pellegrino (Italy), Gerolsteiner (Germany), San Benedetto and Ferrarelle (Italy), Radenska (Slovenia), and Perrier (France).

### Statistical analysis for bottled water study results

Statistical analyses and power calculations were performed in the software R, v. 3.5.1 (R Core Team 2018) from IBM. Mean and standard deviation and the assumption of normality on the log scale were used to calculate the probability that the lower bound of the 95% confidence interval on the geometric mean of the CFU/dose at 15 min post-reconstitution across multiple types of bottled water would equal or exceed the lower acceptance criterion of 4 × 10^8^ CFU/dose for several possible sample sizes. In the simulations, the log10-transformed CFU data were assumed to be Gaussian, and the mean and standard deviation of the data-generating distribution were calculated using the results from a pilot study. We selected a sample size of 6 brands of each type of bottled water for the current study in order to achieve 99% power.

## Results

### Tap water from US and EU

Results from early PXVX0200 product development studies are shown in Table 1. Studies were performed to investigate the viability of CVD 103-HgR lot FA03 in tap water from three US cities (July 2015) and lot 054 in four European cities (May–June 2018).

**Table 1:** PXVX0200 viability when reconstituted in US and EU tap waters (2018).

Source	Arithmetic mean CFU/dose post reconstitution		
	T_0_	15 min	30 min
Miami^a^	6.24 × 10^8^	6.65 × 10^8^	5.82 × 10^8^
San Diego	*1.59* × *10*^*8*^ ± *1.07* × *10*^*7*^	*BLQ*^b^	*BLQ*
Redwood City	6.65 × 10^8^ ± 4.24 × 10^6^	7.88 × 10^8^ ± 2.80 × 10^8^	7.74 × 10^8^ ± 3.76 × 10^8^
Bern	7.99 × 10^8^ ± 3.69 × 10^8^	6.28 × 10^8^ ± 3.60 × 10^8^	5.53 × 10^8^ ± 2.51 × 10^8^
London^c^	1.03 × 10^9^ ± 2.40 × 10^8^	9.82 × 10^8^	8.60 × 10^8^ ± 1.51 × 10^8^
Madrid	6.08 × 10^8^ ± 3.81 × 10^8^	5.50 × 10^8^ ± 3.83 × 10^8^	4.12 × 10^8^ ± 4.26 × 10^8^
Milan	1.10 × 10^9^ ± 1.98 × 10^8^	8.67 × 10^8^ ± 1.69 × 10^8^	8.30 × 10^8^ ± 1.88 × 10^8^

Italicized values are below the PXVX0200 lower potency limit.

^a^Miami sample was tested in singlicate due to availability of sample at the time of the study.

^b^*BLQ* below level of quantification for the CFU assay.

^c^15 min time-point for London sample is derived from a single measurement as the duplicate had no growth, which was attributed to a missed dilution during CFU sample preparation.

Reconstitution of PXVX0200 in San Diego tap water resulted in CVD 103-HgR viability below the lower potency limit of the vaccine (4 × 10^8^ CFU/dose) at time zero, immediately after reconstitution, which then decreased to below the level of quantification for the CFU assay at 15 and 30 min post-reconstitution. PXVX0200 reconstituted in tap waters from Miami and Redwood City remained above the potency specification for at least 30 min following reconstitution.

CVD 103-HgR viability was maintained in all tap water samples from Europe, however one of the duplicate measurements from the Madrid sample at 15 and 30 min post-reconstitution were below the specification (2.79 × 10^8^ and 1.10 × 10^8^ CFU/dose, respectively). The other replicate values for Madrid tap water 15 and 30 min post-reconstitution were within specification (8.20 × 10^8^ and 7.13 × 10^8^, respectively), so the averages for the duplicates were also within specification though the variability in the measurements was high (> 30% relative standard deviation) for these replicates.

PXVX0200 reconstituted in the remaining European tap waters remained above the lower limit of potency for the vaccine for at least 30 min.

CVD 103-HgR active component lot 230 was used during February–March 2019 studies in which San Diego, Redwood City, and Madrid tap waters were re-tested for CVD 103-HgR viability (Table 2) as well as water chemistry analysis.

**Table 2:** PXVX0200 viability when reconstituted in select tap-waters from US and EU (2019).

Source	Arithmetic mean CFU/dose post reconstitution		
	T_0_	15 min	30 min
San Diego	*BLQ*	*BLQ*	*BLQ*
Redwood City	*1.19* × *10*^*8*^ ± *6.80* × *10*^*7*^	*BLQ*	*BLQ*
Madrid	8.23 × 10^8^ ± 2.33 × 10^7^	8.41 × 10^8^ ± 2.97 × 10^7^	7.64 × 10^8^ ± 7.78 × 10^6^

Italicized values are below the PXVX0200 lower potency limit.

*BLQ* below level of quantification.

CVD 103-HgR was not viable in Redwood City tap water samples collected for the 2019 experiments, and Madrid tap water was shown compatible with PXVX0200. CVD 103-HgR was not compatible with San Diego tap water as seen in previous experiments.

### Tap water chemical compound spiking results

Water chemistry panel data on aliquots of the February–March 2019 tap waters from San Diego, Redwood City (RWC), and Madrid were compared, and candidate chemical compounds were identified. We focused on chemical compounds that were by-products of the disinfection process, and chemical compounds found at higher concentration in San Diego and Redwood City tap water compared to Madrid. If the chemical compound was not found in any of the three samples, it was not included (Table 3).

**Table 3:** Water chemistry panel results on Madrid, San Diego, and Redwood City tap waters (2019).

Tap water source	Madrid	San Diego	RWC^a^	Units	EPA MCL
Chlorine (residual)	0.87	1.63	2.15	mg/L	4 mg/L
Copper	–	0.111	0.078	mg/L	1.3 mg/L
Bromodichloromethane	2.84	7.94	3.69	ug/L	Trihalomethane combined limit: 80 ug/L
Bromoform	–	2.45	–	ug/L	
Chlorodibromomethane	–	7.76	–	ug/L	
Fluoride	0.050	0.66	0.79	mg/L	4.0 mg/L

Samples were run in singlicate for all water chemistry analysis.

*MCL* maximum contaminant level.

^a^*RWC* Redwood City.

The results from spiking the chemical compounds into MBGW are shown in Table 4.

**Table 4:** Viability of CVD 103-HgR active component reconstituted in MBGW spiked with tap water chemical compound candidates (2019).

Chemical compound	Concentration (mg/L)	CFU/dose post reconstitution		
		T_0_	15 min	30 min
Sodium hypochlorite (chlorine)	5	6.70 × 10^8^	6.58 × 10^8^	5.56 × 10^8^
	10	5.78 × 10^8^	5.52 × 10^8^	4.89 × 10^8^
	100	5.33 × 10^8^	5.37 × 10^8^	3.86 × 10^8^
Copper	13	BLQ	BLQ	BLQ
	0.078	BLQ	BLQ	BLQ
Bromodichloromethane	0.8	7.38 × 10^8^	6.93 × 10^8^	6.54 × 10^8^
Bromoform	0.8	7.62 × 10^8^	7.09 × 10^8^	6.64 × 10^8^
Chlorodibromomethane	0.8	8.52 × 10^8^	8.41 × 10^8^	7.88 × 10^8^
Fluoride	40	7.64 × 10^8^	6.97 × 10^8^	7.02 × 10^8^

Samples were run in singlicate for all CFU assays for water chemistry analysis.

The CVD 103-HgR active component reconstituted in MBGW spiked with 13 mg/L copper, which is 10 times the MCL allowed in drinking water, was not viable, nor was it viable in 0.078 mg/L MBGW spiked copper solution, which correlated to the lowest concentration of copper observed in the water chemistry panels (Redwood City).

The CVD 103-HgR active component viability was not substantially affected by bromodichloromethane, chlorodibromomethane, bromoform, or fluoride spiked at 10 times the MCL allowed in drinking water.

Chlorine was effectively neutralized by the ascorbic acid in the buffer component, though chlorine at 100 mg/L (25 times the MCL) appears to affect the viability.

### Still and sparkling bottled water from US and EU

Bottled spring and purified bottled water studies (Table 5) were performed on top-selling brands of water in the US in August–October 2016 using CVD 103-HgR active lot 001.

**Table 5:** PXVX0200 viability when reconstituted in bottled spring and purified bottled waters (2016).

Water label	Brand	Arithmetic mean CFU/dose post reconstitution		
		T_0_	15 min	30 min
Bottled spring water	Poland Spring	1.02 × 10^9^ ± 7.07 × 10^6^	1.03 × 10^9^ ± 5.87 × 10^7^	8.12 × 10^8^ ± 1.24 × 10^8^
	Deer Park	1.04 × 10^9^ ± 0.00	9.92 × 10^8^ ± 2.55 × 10^7^	7.84 × 10^8^ ± 7.07 × 10^7^
	Ozarka	1.08 × 10^9^ ± 2.12 × 10^7^	1.03 × 10^9^ ± 7.07 × 10^6^	8.90 × 10^8^ ± 4.38 × 10^7^
	Evian	1.19 × 10^9^ ± 1.41 × 10^7^	1.11 × 10^9^ ± 3.54 × 10^7^	9.93 × 10^8^ ± 2.47 × 10^7^
	Crystal Geyser	1.17 × 10^9^ ± 2.83 × 10^7^	1.21 × 10^9^ ± 7.78 × 10^7^	1.07 × 10^9^ ± 4.95 × 10^7^
	Arrowhead	1.24 × 10^9^ ± 1.41 × 10^7^	1.13 × 10^9^ ± 4.24 × 10^7^	1.07 × 10^9^ ± 3.54 × 10^7^
Purified bottled water	DASANI	1.10 × 10^9^ ± 4.95 × 10^7^	1.17 × 10^9^ ± 3.54 × 10^7^	1.02 × 10^9^ ± 0.00
	Aquafina	1.11 × 10^9^ ± 7.07 × 10^7^	8.98 × 10^8^ ± 2.12 × 10^6^	7.12 × 10^8^ ± 1.06 × 10^7^
	Nestle Pure Life	1.08 × 10^9^ ± 1.41 × 10^7^	1.04 × 10^9^ ± 5.66 × 10^7^	8.83 × 10^8^ ± 2.76 × 10^7^
	Glaceau Smartwater	1.07 × 10^9^ ± 7.07 × 10^6^	8.90 × 10^8^ ± 7.14 × 10^7^	8.03 × 10^8^ ± 5.73 × 10^7^
	Niagara	9.87 × 10^8^ ± 1.89 × 10^8^	8.76 × 10^8^ ± 8.91 × 10^7^	7.07 × 10^8^ ± 1.20 × 10^7^
	Kirkland Select	1.03 × 10^9^ ± 9.19 × 10^7^	8.69 × 10^8^ ± 7.64 × 10^7^	8.48 × 10^8^ ± 2.47 × 10^7^

European still bottled water experiments were performed in May 2019 with CVD 103-HgR active lot 230 and sparkling bottled water experiments were performed in April–July 2020 with lot 076 (Table 6).

**Table 6:** PXVX0200 viability when reconstituted in European still and sparkling natural spring waters (2020).

Water type	Brand	Arithmetic mean CFU/dose post reconstitution		
		T_0_	15 min	30 min
Still bottled water	REWE	6.35 × 10^8^ ± 4.95 × 10^7^	7.05 × 10^8^ ± 3.46 × 10^7^	6.73 × 10^8^ ± 3.54 × 10^7^
	Carrefour	7.43 × 10^8^ ± 4.24 × 10^6^	7.40 × 10^8^ ± 1.84 × 10^7^	6.83 × 10^8^ ± 2.40 × 10^7^
	Volvic	7.22 × 10^8^ ± 4.10 × 10^7^	7.27 × 10^8^ ± 1.91 × 10^7^	7.28 × 10^8^ ± 3.54 × 10^7^
	Acqua Panna	7.15 × 10^8^ ± 1.56 × 10^7^	7.28 × 10^8^ ± 3.68 × 10^7^	6.98 × 10^8^ ± 3.54 × 10^7^
	Henniez	6.79 × 10^8^ ± 1.91 × 10^7^	6.97 × 10^8^ ± 2.12 × 10^6^	6.55 × 10^8^ ± 1.13 × 10^7^
	Evian	6.34 × 10^8^ ± 9.26 × 10^7^	6.29 × 10^8^ ± 8.91 × 10^7^	6.10 × 10^8^ ± 5.80 × 10^7^
Sparkling bottled water	S.Pellegrino	7.83 × 10^8^ ± 1.98 × 10^7^	7.81 × 10^8^ ± 4.03 × 10^7^	6.96 × 10^8^ ± 4.81 × 10^7^
	Gerolsteiner	8.17 × 10^8^ ± 4.45 × 10^7^	7.73 × 10^8^ ± 2.05 × 10^7^	8.26 × 10^8^ ± 3.18 × 10^7^
	San Benedetto	1.05 × 10^9^ ± 0.00	9.77 × 10^8^ ± 2.47 × 10^7^	1.01 × 10^9^ ± 0.00
	Farrarelle	7.04 × 10^8^ ± 6.58 × 10^7^	8.12 × 10^8^ ± 5.73 × 10^7^	8.15 × 10^8^ ± 6.43 × 10^7^
	Radenska	7.83 × 10^8^ ± 2.62 × 10^7^	8.47 × 10^8^ ± 6.15 × 10^7^	8.80 × 10^8^ ± 7.21 × 10^7^
	Perrier	9.96 × 10^8^ ± 3.46 × 10^7^	9.78 × 10^8^ ± 2.69 × 10^7^	9.39 × 10^8^ ± 6.43 × 10^7^

Results show that all tested brands of European still and sparkling natural mineral waters are compatible with PXVX0200.

Statistical analysis of waters that are currently approved for reconstitution of the PXVX0200 vaccine, including spring and purified bottled waters (US) and natural spring water (EU), was performed for each time-point (Table 7).

**Table 7:** Geometric mean CFUs and 95% confidence intervals by type of bottled water over time (2016, 2019).

Post-reconstitution (min)	Spring water—6 brands (US)	Purified water—6 brands (US)	Natural spring water—6 brands (EU)
0	1.12 × 10^9^ [1.03 × 10^9^, 1.21 × 10^9^]	1.06 × 10^9^ [9.98 × 10^8^, 1.12 × 10^9^]	6.86 × 10^8^ [6.40 × 10^8^, 7.35 × 10^8^]
15	1.08 × 10^9^ [1.00 × 10^9^, 1.16 × 10^9^]	9.49 × 10^8^ [8.41 × 10^8^, 1.07 × 10^9^]	7.02 × 10^8^ [6.61 × 10^8^, 7.46 × 10^8^]
30	9.26 × 10^8^ [8.09 × 10^8^, 1.06 × 10^9^]	8.20 × 10^8^ [7.13 × 10^8^, 9.44 × 10^8^]	6.73 × 10^8^ [6.33 × 10^8^, 7.15 × 10^8^[

The combined data for all brands of bottled spring water, purified bottled water, and bottled natural mineral water remain above the lower limit of specification for the PXVX0200 vaccine for at least 30 min.

## Discussion

The aim of this study was to investigate the compatibility of CVD 103-HgR, the active ingredient in the PXVX0200 oral cholera vaccine, with drinking water from different sources in the US and Europe. Tap water quality in developed nations is regulated by law, however chemicals are still common and may be derived from natural sources, disinfection procedures, and plumbing.

The CVD 103-HgR active component was not viable in San Diego tap water, and demonstrated various levels of viability when reconstituted in tap waters from Madrid and Redwood City. One possible cause of the variation is that the waters were collected in different years and during different months. The CVD 103-HgR active component was stable when reconstituted in tap water collected from Redwood City, California, in July 2015, but wasn’t stable when reconstituted in water from the same source collected in February, 2019. Conversely, the viability of CVD 103-HgR active component was inconsistent when reconstituted in tap water collected from Madrid in May/June 2018, but was stable when reconstituted in tap water from the same source collected in February/March 2019. This suggests that there is seasonal variability in tap-water chemistry, which is supported by the literature^17^. Further investigation into the specific chemistry of San Diego and Redwood City tap waters collected in February/March 2019 revealed several contaminants at detectable levels. Spiking studies were performed with these contaminants and it was found that that copper, even at relatively low concentration (0.078 mg/L) substantially affected the viability of CVD 103-HgR active component. The most common source of copper in drinking water is corrosion of plumbing, faucets, and water fixtures^18,19^. Based on these results, tap water should not be used to reconstitute the PXVX0200 cholera vaccine prior to administration.

All bottled water brands tested from the US and European countries were compatible with the CVD 103-HgR active component, including 6 bottled spring and 6 purified bottled water brands in the US, and 12 natural mineral waters (6 still water brands and 6 sparkling water brands) from Europe. The PXVX0200 reconstitution instructions include only bottled spring and purified bottled waters in the US, and still bottled waters in Europe.

These results support the conclusion that tap water should not be used to reconstitute the PXVX0200 vaccine, but instead still bottled spring, purified bottled, and natural mineral waters may be used.
